# Early cancer detection among rural and urban californians

**DOI:** 10.1186/1471-2458-6-194

**Published:** 2006-07-26

**Authors:** Sarah L Blair, Georgia R Sadler, Rebecca Bristol, Courtney Summers, Zanera Tahar, Sidney L Saltzstein

**Affiliations:** 1 Department of Surgery, University of California at San Diego, San Diego, California, USA; 2 University of California at San Diego Undergraduate Education, San Diego California, USA; 3 University of California at San Diego Medical School, San Diego California, USA; 4 Department of Pathology University of California at San Diego, San Diego, California, USA; 5 Department of Preventive Medicine University of California at San Diego, San Diego, California, USA

## Abstract

**Background:**

Since the stage of cancer detection generally predicts future mortality rates, a key cancer control strategy is to increase the proportion of cancers found in the early stage. This study compared stage of detection for members of rural and urban communities to determine whether disparities were present.

**Methods:**

The California Cancer Registry (CCR), a total population based cancer registry, was used to examine the proportion of early stage presentation for patients with breast, melanoma, and colon cancer from 1988 to 2003. Cancer stage at time of detection for these cancers was compared for rural and urban areas.

**Results:**

In patients with breast cancer, there were significantly more patients presenting at early stage in 2003 compared to 1988, but no difference in the percentage of patients presenting with early stage disease between rural and urban dwellers. There were no differences in incidence in early stage cancer incidence between these groups for melanoma patients, as well. In colorectal cancer in 1988, significantly more patients presented with early stage disease in the urban areas (42% vs 34%, p < 0.02). However, over time the rural patients were diagnosed with early stage disease with the same frequency in 2003 as 1988.

**Conclusion:**

This analysis demonstrates that people in rural and urban areas have their breast, melanoma or colorectal cancers diagnosed at similar stages. Health care administrators may take this information into account in future strategic planning.

## Background

Addressing identified pockets of health disparities within various population sub-groups has become a major focus among leading cancer organizations as they work toward achieving their cancer control goals [1,2]. Researchers have identified health disparities among economic groups [3], ethnic groups [4], sexual identity groups [5], socioeconomic groups [6], genders [7] and geographic locations, such as urban verses rural [8,9]. Stage of diagnosis is the single best predictor of cancer morbidity and mortality. This study used data from the California Cancer Registry to test whether differences exist in the stage of cancer diagnosis between rural areas and urban areas. Knowing where differences in stage of cancer diagnosis exist can help health policy makers and educators plan more effective cancer control interventions.

Previous studies have identified significant disparities in stages of diagnosis between people living in rural and urban areas, with a greater incidence of late stage diagnosis generally found in rural areas [10][11]. This has been attributed to limited access to clinics and hospitals with the advanced technology needed to detect cancer in the early stage and may need to travel great distances to receive care [12]. Lack of insurance available to individuals that reside in rural areas is another problem for these patients [13]. In addition, members of rural communities may lack basic cancer information because they do not have the same access to cancer education programs that are offered in urban areas [14]. At least one study suggests that physicians in the rural community may not encourage screening as strongly as physicians living in urban communities [14]. The authors of this study did not interview physicians to find out the cause for this difference but rural women were less likely to undergo a clinical breast exam and be referred for a screening mammogram [14].

While these factors are reported to contribute to a higher rate of late stage cancer detection in rural versus urban areas, a study by McElroy found no significant difference in early detection of cancer between urban and rural communities [15]. He attributed this lack of observed disparity to the fact that the use of technology has expanded, and more women living in rural areas are receiving screening [15]. With these conflicting results in the literature, the aim of this study was to examine whether discrepancies in the rates of diagnosis of early stage cancers could be found among patients in the California Cancer Registry (CCR).

## Methods

In 1988, cancer was made a mandatory reportable disease in California. The California Cancer Registry (CCR) became the repository for the data. New cases are reported by physicians, health facilities, laboratories, and death certificates, and there are penalties for failure to report cases. The actual abstracting of cases is done by certified Cancer Abstracters. Ten Regional Cancer Registries were set up to act as the first-level receiving facility and to provide quality assurance, coordination of reports from multiple sources for the same patient, provide technical help to those reporting cases, and then to report the data to the central registry in Sacramento. At this level, there is further quality assurance, elimination of duplicate entries, clearance with death certificates, and mutual referral of reports of patients actually residing in other states and countries. The CCR is certified by the North American Association of Central Cancer Registries (NAACCR) and currently the entire state is part of NCI's Surveillance, Epidemiology, and End Results Program (SEER). California has the largest population of any single total population-based registry in the world.

With California's very large population (36 million in 2003), CCR is the total population-based registry encompassing the largest population in the world, and probably, the most diverse. Sixteen years of data (2,387,316 individual cancers), with all patient, physician, and institutional identification removed, are available to researchers.

The data set includes demographics of the patient, features of the tumor such as stage and histology, and follow-up information. The data are made available to qualified researchers on a CD. Intercensal population data developed by the California Department of Finance have been supplied by the CCR.

There are no uniformly accepted definitions of "rural" and "urban" for registry data. We have chosen the one used by the California Rural Health Commission [16], using counties as the areas of interest. "Rural" counties are those with a population density of 250 people or less per square mile and no incorporated communities of more than 50,000 people. Using this definition, we identified 28 "rural" counties (population density range 1.56 to 98.2 people/sq. mi.) having a total population of 1,842,118. For an "urban" comparison group, we selected the two counties with the highest population densities (3,488.58 and 17,213.49) people per square mile and which contain 4,476,251 people), rather than those with the largest populations, leaving 28 other counties in a "mixed" category and not further studied. Since we do not have the patients' actual addresses, socioeconomic data and because these, as well as facilities, transportation, effects of educational programs, the role of mobile mammographic facilities, etc., often change with time, we cannot evaluate what part may be played by any of these. Moreover, one must be aware that even in the two groups, there may be county-to-county differences.

Using the above definitions of "rural" and "urban", all of the cases which had the years of diagnosis and Summary Stage of breast cancer, colorectal cancer, and cutaneous malignant melanoma from the years 1988 through 2003 were studied. For each type of cancer, data were tabulated by years of diagnosis and "early" versus "late" stage, using the "EpiInfo6" (CDC and WHO) software. As noted above, there are multiple levels of quality assurance, and the standards of the North American Association of Central Cancer Registries (NAACCR) are all met or exceeded. The tissue diagnoses of the original pathologists are accepted because central review would be a fiscal and operational impossibility. It should be remembered that the patient's therapy is based on the diagnosis by the local pathologist.

Early" stage is defined as a Summary Stage of carcinoma-in-situ or localized tumor, and "late" stage is a Summary Stage of regional or distant disease. Summary Stages are rigorously defined by the NCI's Surveillance, Epidemiology, and End Results Program (SEER), the American Joint Commission on Cancer, and the American College of Surgeons. The definitions of stages differ from one primary site to another. While for clinical purposes it might be preferable to use the TNM System, there are no TNM staging data for the earlier years of CCR for all sites and thus not possible to use the TNM system. Moreover, to allow comparison with earlier registry data as well as data from other registries, the Summary Stage is more useful. When the data exist, cross- tabulations of Summary Stage "localized" against "T1N0M0" show a correlation of over 90%. We do not have data on Breslow stage. Staging data are not available for every case. Finally, while the CCR has information about ethnicity, there are not enough numbers in the non-white rural population to draw any meaningful conclusions to include in this manuscript. Because this study used the above data, it was exempt from the usual methods of Ethical Approval.

## Results

**Figure 1:**
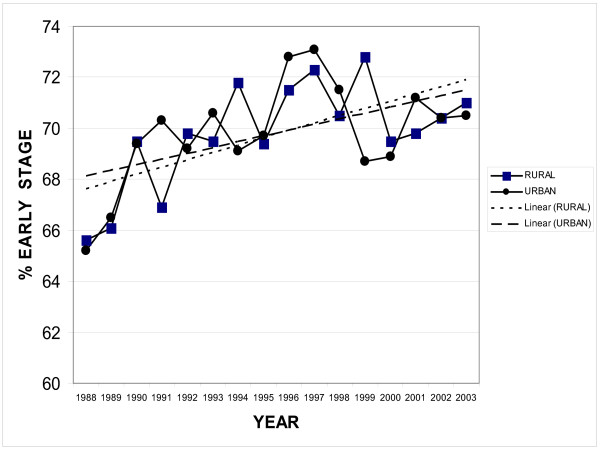
**Early Breast Cancer Rural vs. Urban**. This graph compares the percent of early breast cancers in rural and urban patients over time from 1988 to 2003 in the California Cancer Registry.

Using the above definitions of rural and urban, the 28 rural counties were compared to the two urban counties with the greatest population density. Between 1988 and 2003 there were 59,615 total breast cancers in the registry and 19,428 were in rural patients and 40,187 were in urban patients. There were significantly more patients presenting at early summary stage in 2003 compared to 1988 (71 % vs. 65%, p < 0.007). However, there was no difference in percentage of patients presenting with early summary stage disease between rural and urban dwellers. For example, in 2003, 70% of urban patients presented with early summary stage disease and 69% of urban patients were diagnosed with early summary stage breast cancer. (Figure 1)

**Figure 2:**
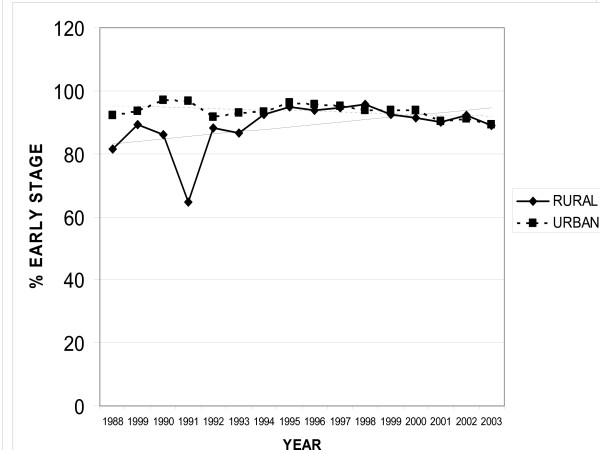
**Cutaneous Melanoma Rural vs. Urban**. This graph compares the percent of early stage melanomas in patients in both urban and rural locations over time from 1988 to 2003 from the California Cancer Registry.

In patients with cutaneous melanoma there were 15,793 total cases in the registry. Rural patients had 5,149 melanomas and urban patients had 10,644; however, there was no difference in summary stage of diagnosis over time. Likewise, there was no difference between rural or urban dwellers in summary stage of diagnosis. In the state of California, an average of 93% of patients present with early summary stage melanoma (Figure 2.)

**Figure 3:**
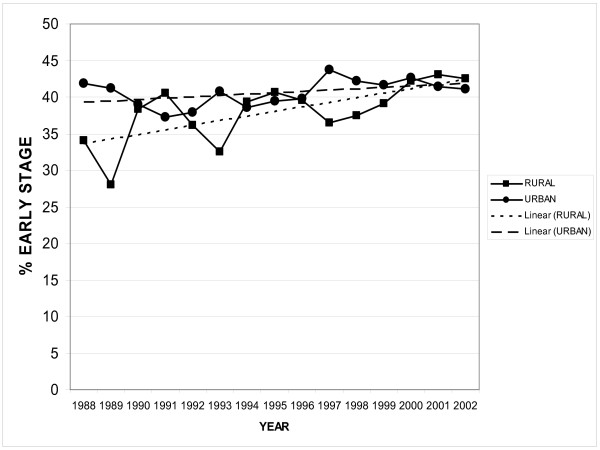
**Colorectal Carcinoma Rural vs Urban**. This graph compares the percent of early stage colorectal cancers in both rural and urban patients over time from 1988 to 2003 from the California Cancer Registry.

For colorectal cancer between 1988 and 2003 there were 26,481 cases recorded. Rural patients had 7,999 cases and urban patients had 18,482 cases. In 1988, significantly more patients presented with early summary stage disease in the urban areas (42% vs 34%, p < 0.02). However, over time the rural patients were diagnosed with early summary stage disease with the same frequency and in 2003, there was no difference in percentage of patients with early summary stage disease (41% vs. 44% P = 0.1, Figure 3).

## Discussion

While health care services are widely available in the United States, access to those services varies widely. Rural-urban differences have been postulated. Financial access to health care is deemed to be a problem for many American who are uninsured or underinsured. Language and cultural barriers can be equally powerful barriers. Health policy planners must carefully consider where to put health care dollars for maximum benefit. Given that detecting early stage cancers is usually better for patients and usually costs less for providers, the central public health goal is to prevent cancers or detect early stage cancers.

In the past, investigators have reported that rural patients had less access to screening modalities and were less likely to present with early stage cancers compared to urban patients. The best studied group is screening women for breast cancer. One recent study has shown a possible reversal in this trend. McElroy et al reported on over 4000 women in Wisconsin diagnosed with breast cancer from 1981–2000. There was an increase incidence of pre-invasive cancer diagnosis and an increase mammography screening in women residing in rural areas [15]. Although this is an important report other data in other parts of the country is needed to confirm theses findings.

Furthermore, Santora el al found in Florida that rural practitioners were more likely to use written breast cancer guidelines compared to urban practitioners [17]. The data from this study confirms McElroy et al.'s finding that there was an increase of diagnosis of early stage breast cancer and likewise there was no difference between rural and urban populations.

In addition, this study confirmed that there were no differences in diagnosis of early stage cancer between these groups for melanoma patients as well. There is little data on influence of location (rural vs. urban) on the incidence of early stage melanoma. Van Durme et al. examined the database from the State of Florida and found that lower median educational attainment but not rural verses urban residence increased the risk of presenting with late-stage disease [18]. This study's data did not find any difference in incidence of earlier stage melanomas from 1988 to 2003. This is similar to results seen by Brackeen et al. in which they found no difference in incidence of thinner melanomas in the state of Texas between 1980 and 2000 despite public education [19].

Lastly, this study did find, like other previous investigators, that there were more locally advanced cancers in rural areas for colorectal cancer in the early period which may reflect less availability of practitioners and technology for screening. However, in the last few years that trend has reversed and there is no difference in incidence of early stage cancers for colorectal cancer as well. Other recent investigators have found that socio-economic status is a more important determinant of early stage disease than rural or urban residence. For example, recent publications concerning these issues in Florida found that socioeconomic status predicted advanced stage disease, but not rural residence and Koka et al. found that in North Dakota distance from a tertiary care center did not seem to be a barrier to early diagnosis of colorectal cancer[20,21].

## Conclusion

At present in California, the California Cancer Registry data demonstrate that people in rural and urban areas have their cancers diagnosed at comparable stages. Health care administrators may take this information into account in future strategic planning.

## Competing interests

The author(s) declare that we have no competing interests.

## Authors' contributions

SLB originated the idea for the manuscript and wrote the majority of the article. RB, CS and AT researched the literature and helped write the background material. GS assembled the team, organized its meetings and assisted in the preparation of the manuscript. SS analyzed the data from the CCR database wrote the methods section and assisted in the preparation of the manuscript.

## Pre-publication history

The pre-publication history for this paper can be accessed here:
